# Education level, mental support, and disease knowledge in relation to adaptability in adult outpatient chemotherapy patients with malignant tumors — a cross‑sectional study

**DOI:** 10.1038/s41598-026-49357-7

**Published:** 2026-04-19

**Authors:** Xiongtao Yang, Hongyan Yan, Chen Tang, Ling Peng, Yuqing Huang, Qiuxi Zhou

**Affiliations:** 1 Department of General Internal Medicine, Sichuan Clinical Research Center for Cancer, Sichuan Cancer Hospital & Institute, Sichuan Cancer Center, University of Electronic Science and Technology of China, Chengdu, China; 2 West China Hospital of Sichuan University, Chengdu, China

**Keywords:** Cancer patients, Outpatient chemotherapy, Adaptability, Influencing factors, Intervention strategies, Patient education, Quality of life

## Abstract

To evaluate the adaptability status of outpatient chemotherapy patients with malignant tumors and identify influencing factors, providing a foundation for developing personalized intervention strategies. This study employed a convenience sampling method to select patients undergoing daytime chemotherapy at a tertiary cancer hospital in Sichuan Province between October and December 2024. Data were collected using a general information questionnaire and the Chinese version of the Outpatient Malignant Tumor Patient Adaptability Scale. Statistical analysis, including univariate and multivariate regression, was conducted using SPSS 26.0. A total of 300 patients were included.The overall adaptability score of the patients was 113.22 ± 13.14, indicating a moderate level of adaptability. Univariate analysis revealed that factors such as age, education level, place of residence, employment status, monthly household income, payment method for medical expenses, chemotherapy cycles, type of mental support, disease care knowledge, and attitude toward the disease significantly influenced adaptability (P < 0.05). Multivariate regression analysis identified education level (β = 1.182, P = 0.019), type of mental support (β = 2.672, P = 0.003), disease care knowledge (β = 10.466, P < 0.001), and attitude toward the disease (β=-9.079, P < 0.001) as key determinants of adaptability. The adaptability of outpatient chemotherapy patients with malignant tumors is influenced by multiple demographic, social, and clinical factors. Enhancing patient education, providing diverse mental support, improving disease knowledge, and fostering positive attitudes are crucial to improving adaptability. These findings can inform the development of targeted interventions to enhance the quality of life and treatment outcomes for cancer patients.

## Introduction

 Cancer remains a leading cause of morbidity and mortality worldwide, with a significant burden on healthcare systems and society^1^. According to the National Cancer Center, China reported 4.82 million new cancer cases and 2.57 million cancer-related deaths in 2022, making it one of the most affected countries globally^2^. The rising incidence and mortality rates, exacerbated by an aging population, underscore the urgent need for effective and accessible cancer treatments^3^.

Daytime chemotherapy has emerged as an innovative medical service model in recent years. This approach allows patients to receive chemotherapy during the day and return home in the evening, reducing the demand for hospital beds, lowering healthcare costs, and improving patient quality of life^4,5^. Despite these advantages, outpatient chemotherapy patients often face unique challenges, including the burden of symptoms, lifestyle disruptions, and the need to adapt to their changing physical and psychological conditions^6,7^.

Outpatient chemotherapy serves as an efficient and convenient treatment model, in which patient adaptability directly influences treatment adherence and quality of life1. However, research exploring the multifactorial determinants of adaptability among adult outpatient chemotherapy patients in China remains limited2. Cross‑sectional studies offer the advantage of efficiently capturing the distribution of exposures and outcomes in a population, clarifying variable associations, and demonstrating high operational feasibility3. This design enables a comprehensive screening of key factors influencing adaptability under limited resources, thereby establishing baseline data for subsequent targeted intervention studies. Hence, this study adopts a cross‑sectional design to conduct this exploration4.

Adaptability, defined as the ability to adjust to new or challenging circumstances, is critical for patients undergoing chemotherapy. Higher adaptability is associated with better psychological well-being, improved adherence to treatment, and enhanced quality of life^8^. However, the adaptability of outpatient chemotherapy patients and its influencing factors remain under-researched, particularly in China. Understanding these factors is essential for developing targeted interventions to support patients during their treatment journey.

This study aims to investigate the adaptability of outpatient chemotherapy patients with malignant tumors and analyze the factors influencing it. Using the Chinese version of the Outpatient Malignant Tumor Patient Adaptability Scale^9^, we sought to provide evidence-based insights to inform clinical practice and policy development. By identifying key determinants of adaptability, this study contributes to the growing body of literature on improving the quality of life and treatment outcomes for cancer patients.

## Methods

### Study design and participants

This cross-sectional study was conducted at Sichuan Cancer Hospital, a high-volume oncology center, from October to December 2024.

### Inclusion criteria

Participants were eligible for inclusion if they met all of the following conditions:

Aged 18 years or older at the time of enrollment.Diagnosed with a malignant tumor and currently receiving outpatient (daytime) chemotherapy at Sichuan Cancer Hospital during the study period (October–December 2024).Had clear consciousness and were able to communicate effectively in Chinese.Were aware of their diagnosis and treatment status.Were willing to participate and provided written informed consent.Completed the questionnaire independently or with minimal assistance and provided logically consistent responses.

### Exclusion criteria

Participants were excluded if they met any of the following conditions:

A documented history of psychiatric disorders or severe cognitive impairment that could interfere with questionnaire completion or informed consent.Experiencing Grade 3 or higher chemotherapy-related adverse events (assessed by the Common Terminology Criteria for Adverse Events, CTCAE Version 5.0) at the time of data collection—these events were defined as severe or medically significant but not immediately life-threatening, resulting in significant functional impairment or requiring intensive medical intervention that precluded the continuation of scheduled outpatient chemotherapy.Acute complications or life-threatening conditions at the time of data collection.Inability to complete the questionnaire due to physical, cognitive, or language barriers.Submission of questionnaires that were incomplete, contradictory, or illogical, as determined during on-site review.

The study was approved by the Medical Research and New Technology Ethics Committee of Sichuan Cancer Hospital (Approval No.: SCCHEC-02–2024-184). All methods were performed in accordance with relevant guidelines and regulations, and informed consent was obtained from all participants prior to their inclusion in the study.

### Sample size estimation

The sample size was determined based on the commonly used simplified criteria for multivariate linear regression analysis—to ensure stable statistical parameter estimation, a minimum of 15 participants per independent variable is required.In this study, 9 candidate independent variables were initially included in the regression model: age, education level, place of residence, monthly household income, payment method for medical expenses, chemotherapy cycles, type of mental support, knowledge of disease care, and attitude toward the disease. According to the above criterion, the minimum required sample size was calculated as follows: Minimum sample size = Number of candidate independent variables × 15 participants/variable = 9 × 15 = 135 participants.To account for potential invalid questionnaires (e.g., incomplete or illogical responses) and missing data, an additional 20% margin was added to the minimum sample size: Adjusted minimum sample size = 135 × 1.20 = 162 participants.Thus, the study required a minimum of 162 participants to meet the statistical analysis requirements.A total of 300 outpatient chemotherapy patients were ultimately included in the final analysis, which substantially exceeded the adjusted minimum sample size (162 participants).

### Data collection tools

#### General information questionnaire

The general information questionnaire was designed to capture both demographic and disease-related information. Demographic variables included age, gender, education level, marital status, employment status, monthly household income, payment method for medical expenses, and place of residence. Disease-related variables included cancer type, cancer staging, duration since diagnosis, and the number of completed chemotherapy cycles.

#### Chinese version of the outpatient malignant tumor patient adaptability scale

The Chinese version of the Outpatient Malignant Tumor Patient Adaptability Scale, adapted from the original scale developed by Keiko et al., was used to measure patient adaptability. The scale consists of six dimensions and 41 items: communication ability, ability to seek better coping strategies, ability to increase certainty, ability to recognize and manage physical changes, ability to adjust goals, and ability to control physical functions. Each item is rated on a 5-point Likert scale, ranging from “completely disagree” (0 points) to “completely agree” (4 points), with higher scores indicating stronger adaptability. The scale has demonstrated good reliability and validity, with a Cronbach’s α coefficient of 0.834, split-half reliability of 0.841, and test-retest reliability of 0.796, making it suitable for this study.

#### Data collection procedures

Trained clinical nurses conducted the data collection process. Participants were informed of the study’s purpose, and written consent was obtained before participation. Nurses provided uniform instructions for completing the questionnaires, which were collected on-site and checked for completeness. Invalid responses were excluded from further analysis.

### Statistical analysis

Data were double-checked for accuracy and entered into Excel before analysis using SPSS 26.0 software. Descriptive statistics were used to summarize demographic and clinical characteristics. Univariate analyses, including one-way ANOVA and independent sample t-tests, were conducted to compare adaptability scores across groups. Multivariate linear regression was performed to identify factors significantly influencing adaptability. Statistical significance was set at *P* < 0.05.

## Results

### General characteristics of the study participants

A total of 300 outpatient chemotherapy patients were included in the study. The majority of participants were female (52.7%), and the mean age was 53.2 ± 12.8 years. Most patients had completed high school education (40.2%), with a smaller proportion having attained college-level education or higher (20.3%). Regarding employment status, 35.6% of participants were employed, while 42.8% were retired. Urban residents accounted for 63.3% of the sample, and most participants relied on medical insurance to cover treatment costs (78.7%).

### Adaptability status of outpatient chemotherapy patients

The overall adaptability score of the participants was 113.22 ± 13.14, indicating a moderate level of adaptability. Among the six dimensions of the adaptability scale, the highest mean scores were observed in “ability to adjust goals” (3.10 ± 0.39) and “ability to control physical functions” (3.08 ± 0.31). Conversely, the lowest scores were recorded in “ability to recognize and manage physical changes” (2.37 ± 0.45) and “ability to seek better coping strategies” (2.51 ± 0.50). These results suggest that while patients generally performed well in adjusting goals and managing physical functions, they faced challenges in adapting to physical changes and finding effective coping strategies (Table 1).

**Table 1:** Adaptability status of outpatient chemotherapy patients with malignant tumors (*n* = 300).

Dimension	Number of items	Total score (mean ± SD)	Mean score per item (mean ± SD)
Overall	41	113.22 ± 13.14	-
Dimension 1: Communication ability	11	32.63 ± 3.87	2.97 ± 0.35
Dimension 2: Ability to seek better coping strategies	11	27.62 ± 5.46	2.51 ± 0.50
Dimension 3: Ability to increase certainty	4	10.98 ± 1.79	2.75 ± 0.45
Dimension 4: Ability to recognize and manage physical changes	6	14.22 ± 4.01	2.37 ± 0.45
Dimension 5: Ability to adjust goals	4	12.39 ± 1.57	3.10 ± 0.39
Dimension 6: Ability to control physical functions	5	15.39 ± 1.54	3.08 ± 0.31

### Univariate analysis of factors influencing adaptability

Univariate analysis identified several factors significantly associated with adaptability (*P* < 0.05), including age, education level, place of residence, employment status, monthly household income, payment method for medical expenses, completed chemotherapy cycles, type of mental support, knowledge of disease care, and attitude toward the disease. Patients aged 41–50 years had the highest adaptability scores, while those over 60 years scored the lowest (*P* < 0.01). Education level also significantly influenced adaptability, with patients who had attained a college degree or higher scoring higher than those with lower levels of education (*P* < 0.001). Urban residents exhibited higher adaptability than rural residents (*P* = 0.002), and employed patients had significantly better adaptability than unemployed or retired participants (*P* = 0.003). Detailed results are shown in Table 2.

**Table 2:** Univariate analysis of adaptability in outpatient chemotherapy patients (*n* = 300).

Variable	Group	Number (%)	Adaptability score (mean ± SD)	F-value	*p*-value
Gender	Male	142 (47.3%)	113.15 ± 13.25	0.007	0.952
	Female	158 (52.7%)	113.28 ± 13.08		
Age	18–25 years	2 (0.6%)	108.50 ± 13.43	4.042	0.001
	26–30 years	5 (1.6%)	113.40 ± 14.67		
	31–40 years	25 (8.3%)	114.52 ± 13.98		
	41–50 years	66 (22.0%)	118.77 ± 8.39		
	51–60 years	109 (36.3%)	112.67 ± 10.91		
	Over 60 years	93 (31.0%)	109.68 ± 16.53		
Education level	Primary School	81 (27.0%)	108.02 ± 13.47	8.534	<0.001
	Middle School	105 (33.0%)	114.02 ± 10.35		
	High School	45 (15.0%)	111.22 ± 15.81		
	College	45 (15.0%)	120.87 ± 10.37		
	Bachelor’s and Above	24 (8.0%)	116.71 ± 14.56		
Place of residence	Urban	163 (54.3%)	115.19 ± 13.19	6.408	0.002
	Rural	103 (34.3%)	109.53 ± 11.12		
	Suburban	34 (11.3%)	114.97 ± 16.20		
Marital status	Single	12 (4.0%)	113.14 ± 13.05	0.173	0.842
	Married	274 (91.3%)	112.75 ± 18.05		
	Divorced/Widowed	14 (4.7%)	115.21 ± 10.54		
Employment status	Employed	32 (10.6%)	119.00 ± 9.45	4.680	0.003
	Unemployed	137 (45.6%)	110.72 ± 12.11		
	On Leave/Sick Leave	41 (13.6%)	116.37 ± 12.17		
	Retired	90 (30.0%)	113.54 ± 15.23		
Monthly household income	< 5000	110 (36.7%)	109.45 ± 12.68	7.453	0.001
	5000–10,000	111 (37.0%)	115.47 ± 12.91		
	> 10,000	79 (26.3%)	115.32 ± 13.06		
Payment method	Out-of-pocket	89 (29.4%)	111.07 ± 10.47	3.932	0.004
	Urban health insurance	31 (10.3%)	121.10 ± 12.03		
	Rural cooperative scheme	12 (4.0%)	108.42 ± 16.27		
	Commercial insurance	2 (0.6%)	114.00 ± 21.21		
	Special outpatient	166 (55.3%)	113.25 ± 13.85		
Cancer type	Respiratory tumors	45 (15.0%)	110.89 ± 15.12	1.493	0.159
	Breast tumors	27 (9.0%)	114.44 ± 13.13		
	Hepatobiliary tumors	3 (1.0%)	113.00 ± 7.21		
	Digestive tumors	75 (25.0%)	111.39 ± 12.21		
	Hematological tumors	1 (0.3%)	115.00		
	Head and neck tumors	9 (3.0%)	116.89 ± 13.25		
	Urological tumors	5 (1.6%)	114.00 ± 19.49		
	Female reproductive tumors	77 (25.6%)	111.96 ± 13.08		
	Other tumors	58 (19.3%)	117.86 ± 11.83		
Duration of cancer diagnosis	< 6 months	143 (47.6%)	111.76 ± 12.95	1.650	0.162
	6 months–1 year	83 (27.6%)	114.55 ± 12.65		
	1–2 years	37 (12.3%)	111.95 ± 14.46		
	2–3 years	19 (6.3%)	118.11 ± 13.38		
	> 3 years	18 (6.0%)	116.22 ± 12.78		
Chemotherapy cycles	≤ 2	78 (26.0%)	109.05 ± 14.98	9.097	<0.001
	3–5	140 (46.7%)	112.95 ± 11.77		
	≥ 6	82 (27.3%)	117.66 ± 12.23		
Shortest interval between two visits	Daily	2 (0.6%)	121.50 ± 16.26	0.276	0.948
	1–3 days	1 (0.3%)	111.00 ± 0		
	4–7 days	21 (7.0%)	113.24 ± 13.36		
	10 days–2 weeks	44 (14.6%)	111.95 ± 14.47		
	3 weeks–1 month	228 (76.0%)	113.34 ± 12.93		
	2–3 months	3 (1.0%)	115.33 ± 17.01		
	Other	1 (0.3%)	121.0 ± 0		
Longest interval between two visits	Weekly	7 (2.33%)	110.00 ± 16.72		
	Every 2 weeks	30 (10%)	114.43 ± 14.49		
	Every 3 weeks–1 month	240 (80%)	113.01 ± 12.78		
	Every 2–3 months	23 (7.67%)	114.83 ± 14.53		
Types of mental support	One type	52 (17.3%)	107.60 ± 13.47	8.694	<0.001
	2–3 types	146 (48.6%)	112.84 ± 13.01		
	≥ 4 types	102 (34.0%)	116.64 ± 12.18		
Knowledge of disease and care	None	25 (8.3%)	95.92 ± 14.78	43.516	<0.001
	Partial	246 (88.0%)	113.60 ± 11.36		
	Full	29 (9.7%)	124.93 ± 10.78		
Attitude toward disease	Optimistic	92 (30.67)	112.04 ± 10.12		
	Mood varies with condition	204 (68%)	110.40 ± 11.94		
	Constantly depressed	4 (1.33%)	77.75 ± 13.14		

### Multivariate analysis of factors influencing adaptability

Multivariate regression analysis identified four key predictors of adaptability: education level (β = 1.182, *P* = 0.019), type of mental support (β = 2.672, *P* = 0.003), knowledge of disease care (β = 10.466, *P* < 0.001), and attitude toward the disease (β=−9.079, *P* < 0.001). Higher education levels and diverse types of mental support were positively associated with better adaptability. Comprehensive knowledge of disease conditions and care was the strongest positive predictor, highlighting the importance of health education. In contrast, a negative attitude toward the disease was associated with lower adaptability, underscoring the need for psychological interventions to foster optimism and resilience among patients (Tables 3 and 4).

**Table 3:** Variable assignment.

No.	Included factors	Assignment method
X_1_	Age	18–25 years = 1, 26–30 years = 2, 31–40 years = 3, 41–50 years = 4, 51–60 years = 5, Above 60 years = 6
X_2_	Education level	Primary School = 1, Middle School = 2, High School = 3, College = 4, Bachelor’s or Higher = 5
X_3_	Place of residence	Urban = 1, Rural = 2, Suburban = 3
X_4_	Monthly household income	≤ 5000 = 1, 5000–10,000 = 2, Above 10,000 = 3
X_5_	Payment method	Out-of-pocket = 1, Urban Health Insurance = 2, Rural Cooperative Medical Scheme = 3, Commercial Insurance = 4, Special Outpatient = 5
X_6_	Completed chemotherapy cycles	≤ 2 = 1, 3–5 = 2, ≥ 6 = 3
X_7_	Types of mental support	Only One Type = 1, 2–3 Types = 2, ≥ 4 Types = 3
X_8_	Knowledge of disease and care	No Knowledge = 1, Partial Knowledge = 2, Full Knowledge = 3
X_9_	Attitude toward disease	Optimistic = 1, Mood Changes with Condition = 2, Constantly Depressed = 3, Severely Depressed = 4

**Table 4:** Multivariate regression analysis of adaptability in outpatient chemotherapy patients.

Independent variable	Regression coefficient (B)	Standard error (SE)	Standardized coefficient (β)	95% confidence interval (CI)	t-value	*p*-value
Constant	98.998	4.614	-	90.012–107.984.012.984	21.458	-
Education level	1.182	0.503	0.113	0.195–2.169	2.351	0.019
Types of mental support	2.672	0.882	0.142	0.941–4.403	3.030	0.003
Knowledge of disease and care	10.466	1.512	0.338	0.215–0.461	6.923	<0.001
Attitude toward disease	−9.079	1.306	−0.335	−11.648–6.510	−6.952	<0.001
Model fit indices	-	-	-	Adjusted R² = 0.423	F = 38.65	<0.001

## Discussion

This study found that the overall adaptability score of outpatient chemotherapy patients was moderate (113.22 ± 13.14), with notable variations across the six dimensions. Patients demonstrated strong abilities in adjusting goals and controlling physical functions, but struggled in recognizing and managing physical changes as well as seeking better coping strategies. These findings align with previous research indicating that cancer patients often develop resilience in managing physical symptoms but face psychological challenges when adapting to uncertainties^8,10^. Addressing these weaker areas through targeted interventions, such as coping strategy workshops and emotional resilience programs, could significantly enhance patient adaptability.

The results highlighted several key factors influencing patient adaptability, including education level, mental support, disease-related knowledge, and attitude toward the disease^11^.

Higher education was positively associated with better adaptability (β = 1.182, *P* = 0.019). Educated patients are more likely to access and understand health-related information, enabling them to adopt effective coping strategies^12^. This underscores the importance of tailoring health education programs to meet the needs of patients with lower educational levels, potentially through simplified materials or one-on-one consultations.

The type and diversity of mental support significantly influenced adaptability (β = 2.672, *P* = 0.003). Patients receiving multiple forms of mental support showed higher adaptability, consistent with studies emphasizing the critical role of psychological and social support in managing cancer-related stress^13,14^. Clinical practices should integrate family counseling, peer support groups, and professional therapy to ensure comprehensive mental support for patients.

Disease-related knowledge emerged as the strongest positive predictor of adaptability (β = 10.466, *P* < 0.001). Patients who fully understood their disease and care options demonstrated greater confidence and adaptability, consistent with findings in other oncology studies^15,16^. This highlights the need for systematic health education initiatives, including multimedia resources and interactive sessions, to empower patients with actionable knowledge.

A negative attitude was strongly associated with reduced adaptability (β=−9.079, *P* < 0.001), emphasizing the profound impact of psychological factors on treatment outcomes. Patients with a positive outlook tend to approach treatment challenges with greater resilience, as shown in prior research^17^. Psychological interventions, such as cognitive-behavioral therapy and mindfulness programs, should be routinely offered to foster optimism and emotional stability.

The findings of this study offer valuable insights for improving clinical practices. Tailored interventions should prioritize patients with lower education levels, limited mental support, or negative attitudes toward their disease^18^. Comprehensive health education and mental health support systems should be integrated into routine cancer care to address these challenges effectively.

This study has several limitations. The convenience sampling method and single-center design may limit the generalizability of the findings. Future research should employ multicenter, large-sample studies to validate these results and improve representativeness. Additionally, longitudinal studies are needed to explore changes in patient adaptability over time, providing insights into the long-term impact of interventions.

Further studies should explore the interplay between demographic, psychological, and clinical factors affecting adaptability. The development of standardized adaptability assessment tools and tailored intervention programs could enhance the quality of care for outpatient chemotherapy patients.

The findings of this study provide actionable insights for enhancing outpatient chemotherapy care. By identifying education level, mental support, disease knowledge, and attitude toward illness as key predictors of adaptability, clinicians can implement more personalized and holistic intervention strategies. For instance, patients with lower education levels may benefit from simplified educational materials and one-on-one guidance. Integrating multiple types of mental support-such as psychological counseling, peer groups, and family support-into routine care can address emotional needs and strengthen resilience. Furthermore, structured health education programs should be designed to improve patient understanding of disease and treatment, ultimately empowering patients to actively participate in their care. Promoting positive attitudes through targeted psychosocial interventions may also enhance adaptability, potentially improving treatment adherence and quality of life.

Several limitations should be noted. First, the study utilized a convenience sampling method at a single tertiary cancer center, which may limit the generalizability of the findings to broader populations. Future research should consider multicenter studies with larger and more diverse samples to validate these results. Second, the cross-sectional design precludes the assessment of causality or changes in adaptability over time. Longitudinal studies are warranted to explore the dynamic nature of patient adaptability throughout the treatment trajectory. Lastly, while self-reported measures are practical, they may be subject to recall and social desirability biases, highlighting the need for future studies to incorporate objective or mixed-method assessment tools.

### Limitations and future directions

This study has several limitations. The convenience sampling method and single-center design (tertiary cancer hospital) may limit the generalizability of the findings, as our sample overrepresents urban (63.3%) and insured (78.7%) patients. Future research should employ multicenter, large-sample studies including patients from community hospitals and rural areas to improve representativeness. Additionally, the cross-sectional design precludes the assessment of causality or changes in adaptability over time—longitudinal studies are needed to track adaptability trajectories and evaluate the long-term impact of interventions. Lastly, while self-reported measures are practical, they may be subject to social desirability bias; future studies should incorporate objective indicators (e.g., treatment adherence rates, healthcare utilization) and mixed-methods (e.g., semi-structured interviews) to triangulate results.

Further research should explore the interplay between demographic, psychological, and clinical factors using structural equation modeling, based on the theoretical frameworks integrated in this discussion. The development of standardized adaptability assessment tools and tailored intervention programs for specific subgroups (e.g., rural patients, low-education groups) could enhance the quality of care for outpatient chemotherapy patients in China.

## Conclusion

This study identified the current status and key influencing factors of adaptability in outpatient chemotherapy patients with malignant tumors, revealing a moderate overall adaptability level. Education level, type of mental support, knowledge of disease care, and attitude toward the disease were significant factors affecting adaptability. Tailored interventions, such as enhancing education, providing diverse mental support, and fostering positive attitudes, can significantly improve patient adaptability, quality of life, and treatment outcomes. However, as this study was a single-center, small-sample investigation, its generalizability may be limited. Future research should involve multicenter, large-sample studies and long-term follow-up to validate and refine these findings, providing a more robust basis for supporting chemotherapy patients’ adaptability.

## Data Availability

The datasets generated and analyzed during the current study are available from the corresponding author, Qiuxi Zhou (qiuxi_zhou@126.com), on reasonable request.
